# Language, Speech, and Oral Motor Performance in Children With Developmental Coordination Disorder: A Systematic Review

**DOI:** 10.1111/1460-6984.70117

**Published:** 2025-09-01

**Authors:** Anna Fäldt, Evelien D'haeseleer, Amy De Roubaix

**Affiliations:** ^1^ Child Health and Parenting (CHAP), Department of Public Health and Caring Sciences Uppsala University Uppsala Sweden; ^2^ Ghent University Ghent Belgium

**Keywords:** developmental coordination disorder (DCD), language, neurodevelopmental disorders, oral motor development, speech

## Abstract

**Background:**

Developmental coordination disorder is a neurodevelopmental disorder characterised by motor difficulties that significantly and persistently impact activities of daily living and participation. It has been suggested that children with (probable) developmental coordination disorder (pDCD) experience challenges in the domain of language, speech, and oral motor development.

**Aim:**

This systematic review provides an overview of recent studies assessing challenges in these domains in children with (p)DCD.

**Methods:**

A systematic search was performed in PubMed, Web of Science, EMBASE, and CINAHL, including all peer‐reviewed articles published since January 2002 and up to November 2023 reporting on language, speech, or oral motor performance in children with (p)DCD assessed by standardised instruments.

**Main Contribution:**

A total of fourteen papers were included. The evidence suggests a higher prevalence of speech, language, and oral motor difficulties in children with pDCD.

**Conclusion:**

More high‐quality research, preferably longitudinal, is necessary to examine the prevalence of language, speech, and oral motor difficulties in children with DCD. The review highlights the heightened speech, language, and oral motor challenges faced by children with pDCD.

**WHAT THIS PAPER ADDS:**

*What is already known on this subject*

- Developmental coordination disorder impacts the lives of many children. Parents report that children with developmental coordination disorder also may have challenges in language, speech, and oral motor development.

*What this paper adds to the existing knowledge*

- This study suggests a high prevalence of speech, language, and oral motor difficulties in children with developmental coordination disorder based on standardised measures or diagnosis.

*What are the potential or actual clinical implications for this work?*

- Clinicians should be aware of a large co‐occurrence of developmental coordination disorder and language, speech, and oral motor difficulties to facilitate interventions for the child's difficulties.

## 1 Introduction

Developmental coordination disorder (DCD) is a neurodevelopmental condition defined by four diagnostic criteria in the Diagnostic and Statistical Manual of Psychiatric Disorders (DSM‐5‐TR). DCD is marked by early‐onset (criterion C) motor coordination difficulties (criterion A) affecting daily living activities and participation (criterion B) (American Psychiatric Association Publishing 2022). These difficulties are evident from a young age and cannot be explained by other cognitive, visual, or physical conditions (criterion D). When, in research settings, one or more diagnostic criteria have not been evaluated, it is preferred to use the term “probable DCD” (pDCD) (Smits‐Engelsman et al. 2015). DCD often co‐occurs with other neurodevelopmental conditions such as Attention Deficit Hyperactivity Disorder (ADHD), Autism Spectrum Disorder (ASD), and learning disorders (American Psychiatric Association Publishing 2022). International guidelines (Blank et al. 2019) recommend that a DCD diagnosis be based on a standardised motor assessment and parent, teacher, and/or self‐reports. Furthermore, standardised questionnaires may provide additional support, especially for the evaluation of diagnostic criterion B. A medical doctor must also be involved to rule out other medical conditions that could explain the motor difficulties. The impact of DCD extends beyond the motor domain, often leading to low self‐esteem, mental health issues, and social withdrawal (Zwicker et al. 2013). Children may avoid activities, resulting in fewer friendships and increased isolation (Missiuna et al. 2007). Families also face reduced social participation, emotional strain, and financial hardship, as parents often adjust careers to meet their child's needs (De Roubaix et al. 2025). A subset of children with DCD also experience challenges in speech, language, and oral‐motor development. Even in the first year of life, caregivers and clinicians may observe challenges related to oral motor skills required for breast or bottle feeding in children with DCD (Pless et al. 2001; Hitchcock et al. 2020; Wolthuis‐Stigter et al. 2017). Moreover, one in five children with DCD has experienced early feeding difficulties (Gurevitz 2019). Infants presenting with such feeding difficulties (e.g., poor coordination of sucking, swallowing and breathing or abnormal tongue‐jaw movements) appear to be at increased risk of later being diagnosed with DCD (Rinat et al. 2022). Celletti et al. (2015) proposed that children with DCD may exhibit poor tongue coordination, manifesting as an atypical swallowing pattern. As they grow older, articulation, speech, or language difficulties also seem to become more apparent to parents (De Roubaix et al. 2023; Pless et al. 2001).

When language difficulties are sufficiently severe to impair everyday communication and learning and cannot be explained by another biomedical condition (e.g., brain injury, genetic conditions like Down syndrome, or sensorineural hearing loss), they may meet the criteria for a diagnosis of developmental language disorder (DLD) (Bishop et al., 2017). Similarly, a diagnosis of Speech Sound Disorder (SSD) can be provided when the child experiences severe difficulties in the perception, motor production, or phonological representation of speech sounds and speech segments, which interfere with speech intelligibility. SSD are differentiated into SSD of unknown origin or SSD associated with a diagnosed medical condition and/or underlying deficit, and further differentiated into motor difficulty affecting articulation or a linguistic difficulty affecting phonology (Stringer et al. 2024). DLD has been shown to have a negative effect on behaviour and psychological well‐being throughout the lifespan (Rescorla et al. 2007; McAllister 2016). When DLD co‐occurs with SSD, the combined impact on development can be substantial, often manifesting in difficulties with reading, writing, and the acquisition of mathematical skills. Additionally, this comorbidity may contribute to a negative attitude toward school and learning more broadly (McCormack et al. 2009).

Despite its negative consequences, DLD often remains undetected (Norbury et al. 2016; Tomblin et al. 1997) due to limited public awareness and its nature as an invisible and hidden disorder. Additionally, age‐appropriate phonological abilities may potentially mask underlying language disorders (McGregor 2020). Nevertheless, it is important to identify speech, language, and oral motor difficulties in children with DCD as the co‐occurrence of DCD and DLD may further exacerbate mental health challenges across the lifespan (Farmer et al. 2016; Cranwell et al. 2015; Larson et al. 2011; Flapper and Schoemaker 2013). Some studies also suggest that these children may compensate for their motor difficulties through well‐developed spoken language skills (Smits‐Engelsman et al. 2013). Importantly, many recommended task‐oriented therapy programs, such as Cognitive Orientation to Daily Occupational Performance, rely heavily on verbal ability (Polatajko et al. 2001). Therefore, when additional speech and/or language challenges are present, alternative intervention approaches and supplementary support from a speech‐language therapist may be necessary. In efforts to prevent secondary consequences on mental health, speech and language interventions have been proposed to improve outcomes and help redirect potentially adverse developmental trajectories (Rinaldi et al. 2021; Novak and Morgan 2019; Sciberras et al. 2014; Toseeb et al. 2021; Fisher 2021).

Given the high prevalence of DLD and SSD in children with DCD, and the significant impact these conditions can have on the child's development, it is essential for clinicians to consider the potential co‐occurrence of DLD with DCD. Previous studies have described a high rate of motor difficulties among children with DLD (Hill 2001). However, the relationship between SSD, DLD, and oral motor difficulties in children with DCD is yet to be explored. A deeper understanding of this relationship could support clinical decision‐making and inform public health and educational policy regarding service provision. This study aims to examine the co‐occurrence of (p)DCD with SSD, DLD, or oral motor difficulties in children up to 12 years, using standardised assessments and formal diagnoses of DLD and SSD.

## 2 Methods

This review follows the Preferred Reporting Items for Systematic Reviews and Meta‐Analysis (PRISMA) statement (Page et al. 2021). The review protocol was pre‐registered on PROSPERO (https://www.crd.york.ac.uk/prospero/; registration number CRD42022304587).

### 2.1 Search Strategy

The PICO framework was used with: (1) Population: Children up to 12 years identified with (probable) DCD considering at least one diagnostic criteria described in the DSM; (2) Interest: speech, language, or oral motor performance; (3) Comparison: the DCD group could be compared to typically developing controls, but studies without a control group were also included; and (4) Outcome: only standardised measures of speech, language, or oral motor performance or diagnoses of speech‐language conditions. On 21 December 2022, the following electronic databases were systematically searched: MEDLINE (PubMed), Web of Science, EMBASE (Embase.com), and CINAHL (EBSCOhost). The search was repeated on 14 November 2023 to retrieve any newly published relevant data. The full search strategies can be consulted on PROSPERO.

### 2.2 Selection Criteria

Articles were double‐screened by two authors independently using the Rayyan software (Ouzzani et al. 2016) and had to fulfil the following criteria for inclusion in the review: (1) publication in a peer‐reviewed journal; (2) publication date in or after 2002. Children with co‐occurring conditions or prematurity were not excluded. The inclusion of articles in or after 2002 aims to prioritise the latest and most relevant literature. Both children with a clinical diagnosis of DCD and children with pDCD (when one or more diagnostic criteria have not been evaluated) were included. Studies were excluded if (1) the purpose was to investigate motor performance in a group of children with speech‐language problems, (2) the outcome was related to writing, reading, and dysphagia, or (3) no standardised assessment instrument was used. Discrepancies were discussed in every phase.

### 2.3 Evaluation of Methodological Quality

Included studies were assessed for quality using the Standard Quality Assessment Criteria (QualSyst) (Kmet et al. 2004) by two independent authors. For each study, 14 items were scored as yes (2), partial (1), no (0) or not applicable. Discrepancies in ratings between authors were discussed. Total scores were recalculated as a percentage, only including the applicable answers to enhance comparison.

### 2.4 Data Extraction and Management

Using a standardised extraction spreadsheet in Excel, one author independently extracted the relevant data, which a second author later verified. Details encompassing study design, sample characteristics, outcome measures, and results for the typical development (TD) and pDCD group were extracted from each article. When data were absent, authors were contacted for supplementation, although none were able to provide the requested information.

## 3 Results

**FIGURE 1:**
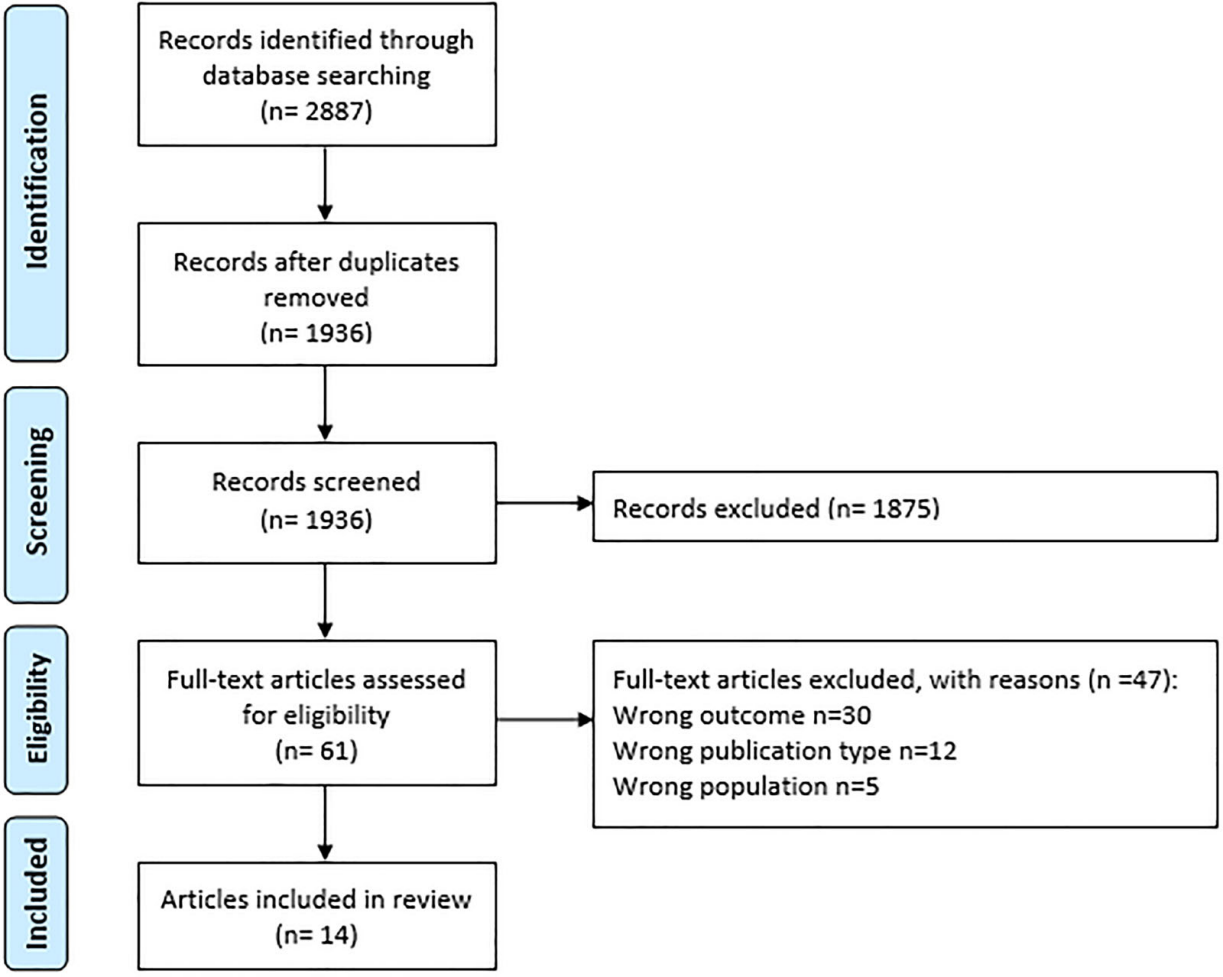
PRISMA flow diagram summarising the search process.

The systematic searches initially identified 2887 articles, reduced to 1936 after deduplication (Figure 1). Next, title and abstract screening resulted in the exclusion of 1875 records, leaving 61 papers for full‐text evaluation. Ultimately, 14 articles met the eligibility criteria for inclusion.

### 3.1 Study Characteristics

Study characteristics can be consulted in Table 1. This systematic review comprised 14 articles published in peer‐reviewed journals between 2003 and 2022. There appears to be a sample overlap between the studies conducted by Cheng et al. (2009, 2022), Dyck and Piek (2010), and Wisdom et al. (2007). Across all included articles, a total of 8789 individuals were included in this systematic review, of whom 843 were identified as having (p)DCD. Half of the articles included children with a clinical diagnosis of DCD (Celletti et al. 2015; Farmer et al. 2016; Flapper and Schoemaker 2013; Gaines et al. 2008; Mirabella et al. 2017; Rodger et al. 2007; Ho and Wilmut 2010), while the others included research‐identified children with pDCD, based on the assessment of one (Archibald and Alloway 2008), two (Cheng et al. 2009, 2022; King‐Dowling et al. 2015) or three diagnostic criteria (Dyck and Piek 2010; Wisdom et al. 2007; Lingam et al. 2010). All studies employed cross‐sectional research designs. Nine articles included a comparison group consisting of typically developing children (Archibald and Alloway 2008; Cheng et al. 2009, 2022; King‐Dowling et al. 2015; Lingam et al. 2010; Mirabella et al. 2017; Ho and Wilmut 2010) or children with poor motor coordination who did not meet criteria for DCD (Dyck and Piek 2010; Wisdom et al. 2007). Four studies (Celletti et al. 2015; Farmer et al. 2016; Flapper and Schoemaker 2013; Rodger et al. 2007) employed a descriptive approach without including a comparison group. Additionally, one case study by Gaines was included (Gaines et al. 2008).

The study quality appraisal can be consulted in Table 2. The average quality rating was 70%, with two high‐quality studies scoring above 80% (Cheng et al. 2009; Lingam et al. 2009). Nine studies fell within the moderate‐quality range, ranging between 60% and 80% (Archibald and Alloway 2008; Celletti et al. 2015; Cheng et al. 2022; Dyck and Piek 2010; Farmer et al. 2016; Gaines et al. 2008; Ho and Wilmut 2010; Mirabella et al. 2017; Wisdom et al. 2007). Three studies, conducted by Flapper and Schoemaker (2013), King‐Dowling et al. (2015), and Rodger et al. (2007), were categorised as low‐quality, scoring below 60%.

**TABLE 1:** Risk of bias assessment with QualSyst.

Criteria	Archibald and Alloway (2008)	Celletti et al. (2015)	Cheng et al. (2009)	Cheng et al. (2022)	Dyck and Piek (2010)	Farmer et al. (2016)	Flapper and Schoemaker (2013)	Gaines et al. (2008)	Ho and Wilmut (2010)	King‐Dowling et al. (2015)	Lingam et al. (2010)	Mirabella et al. (2017)	Rodger et al. (2007)	Wisdom et al. (2007)
1. Question/objective sufficiently described?	2	2	2	1	1	2	1	2	2	2	2	2	1	2
2. Study design evident and appropriate?	2	2	2	2	2	2	2	1	2	2	2	2	1	2
3. Method group selection described and appropriate?	1	2	1	1	1	1	2	2	2	2	2	2	2	2
4. Group characteristics sufficiently described?	1	2	2	2	2	2	2	2	1	1	2	1	2	2
5. Random allocation described?	NA	NA	NA	NA	NA	NA	NA	NA	NA	NA	NA	NA	NA	NA
6. Blinding of investigators reported?	0	0	0	0	NA	0	0	0	0	0	NA	NA	0	0
7. Blinding of subjects reported?	NA	NA	NA	NA	NA	NA	NA	NA	NA	NA	NA	NA	NA	NA
8. Outcomes measures well defined and robust?	1	1	2	2	1	1	1	2	2	1	2	2	2	2
9. Sample size appropriate?	1	1	2	2	1	2	1	1	1	2	2	1	1	1
10. Analytic methods described and appropriate?	1	2	2	2	2	1	1	2	2	1	2	2	1	2
11. Estimates of variance reported?	2	0	2	2	2	0	0	NA	2	0	2	2	0	2
12. Controlled for confounding variables?	0	0	1	2	0	0	0	0	0	0	2	0	0	0
13. Results reported in sufficient detail?	2	1	2	2	2	2	1	2	2	0	2	2	2	2
14. Conclusion supported by results?	2	2	2	1	2	2	2	1	2	2	2	1	1	1
Total score (percentage)	63	63	83	79	73	63	54	68	75	54	100	77	54	75

**TABLE 2:** Included articles discussing language, speech and oral motor performance.

Article	*N* DCD/total	DCD identification	Mean age	Language measurements and TD or normative scores	Outcome DCD group
Archibald and Alloway (2008)	11/33	No clinical DCD diagnosis DSM‐IV‐R criteria: A: M‐ABC Pc <15c <15 B: Not assessed C: Not assessed D: Not assessed, but IQ was assessed (All within average range or higher (SS ≥ 85 Raven's Coloured Matrices. None of the children were diagnosed with ADD, ADHD, autism spectrum disorder or hearing impairment.	DCD: 8y11m ± 1.43 (6y11m to 11y0m) TD: 9y3m ± 1.36 (7y0m to 11y1m)	**CELF‐UK‐3** (Mean = 10 ± 3) Recalling Sentences TD ScSc 9.18 ± 2.68 Word structure TD: not reported **BPVS‐2** (Mean SS = 100 ± 15) TD: SS 109.27 (11.25) **TROG** (Mean SS = 100 ± 15) TD: SS 107.36 (6.45) **GFTA‐2** (mean SS 100 ± 15) TD mean SS = 103.18 ± 6.11 **CNRep** (Norms: 16th percentile → score <22; based on age‐matched controls) TD mean Pc = 27.09 ± 5.338 **Articulation rate** (mean *n* of syllables per second) (Norms: 16th percentile → 5.37 syllables per second; based on age‐matched controls) TD mean *n* = 6.17 (0.79)	*DCD children do not have lower scores on CELF, BVPS, and TROG compared to TD. However, the average score on the CELF subtest of ‘Recalling Sentences’ was below average for the DCD group, indicating expressive language deficits. DCD children had sig. lower scores on CNRep and demonstrated a sig. slower articulation rate compared to TD children. There was no difference in GFTA‐2*. **CELF‐UK‐3** Recalling Sentences: ScSc 6.91 ± 2.77 (n.s.) (4/11 < average) Word structure: ScSc 8.09 ± 2.70 (n.s.) (4/11 < average) **BVPS‐2** SS 100.91 ± 9.18 (n.s.) **TROG** SS 97.55 ± 10.03 (n.s.) (36% below average) **GFTA‐2** DCD mean SS = 95.18 ± 7.69 (n.s.) (1 child < average) **CNRep** DCD mean Pc = 22.36 ± 3.64 (*p* = 0.03) (5/11 below average) **Articulation rate** DCD mean n = 5.62 ± 0.79 (*p* = 0.047) (3/11 < average)
Celletti et al. (2015)	41/41	Clinical DCD diagnosis DSM‐IV criteria: A: M‐ABC B: Not assessed C: Not assessed D: No presence of any neurological, rheumatic, and metabolic disease (evaluated by neurologist, psychiatrist). IQ below 70 was excluded. 19 also had generalised joint hypermobility.	DCD: 8y ± 3y	**LCT** **PPVT** **BNT** **BST** **Memorial‐training test for the assessment of reading and comprehension skills**	*Many children with DCD also seem to have language disorders including narrative difficulties. Children with DCD and generalised hypermobility seem to have more narrative difficulties compared to children with DCD without generalised hypermobility*. 28/41 (68%) language disorders (DCD with hypermobility ns compared to DCD not hypermobile) 12/41 (29%) expressive language disorder (DCD with hypermobility ns compared to DCD not hypermobile) 5/41 (12%) phonological language disorder (DCD with hypermobility ns compared to DCD not hypermobile) 11/41 (27%) receptive/expressive language disorder (DCD with hypermobility ns compared to DCD not hypermobile) 21/41 (51%) narrative difficulties (DCD with hypermobility sig > than DCD not hypermobile)
Cheng et al. (2009)	45/363	No clinical DCD diagnosis DSM‐IV criteria: A: M‐ABC 1.25 SD below the group mean B: not assessed C: not assessed D: No neurological, musculoskeletal, cardiopulmonary, or mental impairments detected. Mean IQ for DCD children = 96.2 ± 14.57 on C‐TONI.	5–6y	**C‐PPVT‐R** **Language Ability assessment for preschoolers** **Composite speech/language tests**	*Weak but sig. correlation M‐ABC and each of the speech and language tests. DCD children were 3x more likely to receive a diagnosis of DSLD*. **C‐PPVT‐R**: r = 0.146, *p* < 0.01 **Language Ability Assessment for Preschoolers**: r = 0.183, *p* < 0.01 **Composite Speech/Language Test for Preschoolers**: r = 0.212, *p* < 0.01 Comorbid DCD + DSLD = 1.65% (Children with DCD are 3x more likely to receive a diagnosis of DSLD than TD children)
Cheng et al. (2022)	64/744	No clinical DCD diagnosis DSM‐IV criteria: A: M‐ABC‐2 Pc <16 B: not assessed C: not assessed D: No neurological, musculoskeletal, and cardiopulmonary system impairments. Children with lower IQ on C‐PPVT‐R (Pc<10) or C‐TONI 3 (SS≤70) were excluded. Mean SS 105.03 ± 11.10 on C‐PPVT‐R and mean SS 89.30 ± 13.37 on C‐TONI‐3.	6.08y ± 0.45 (5–6y)	**C‐PPVT‐R** (mean SS = 100 ± 15) TD: SS 110.22 ± 16.08 **PAT‐C** 14.22% of TD children have PA deficit. TD Total: 14.55 ± 4.65 TD Onset 4.97 ± 1.96 TD Rhyme 5.10 ± 1.95 TD Tone 4.47 ± 2.17 **RAN test** 11.56% of TD children have RAN deficit. TD Total = 55.29 s ± 13.83 TD Digit naming 37.08 s ± 11.74 Colour naming 65.14 s ± 20.70 Object naming 63.73 s ± 15.86	*DCD group performed sig. worse on C‐PPVT‐R, PAT‐C (‘total’ and ‘onset’) and RAN test, but not on PAT‐C (‘Rhyme’ and ‘Tone’) demonstrating poorer phonological awareness and slower naming. However, scores on C‐PPVT‐R are still within average. DCD children were 2.5x more likely to have phonological awareness deficits and 3.5x more likely to have a Rapid Automatic Naming deficit*. **C‐PPVT‐R** SS 105.3 ± 11.10 (*p* = 0.001) **PAT‐C** 29.69% of DCD children have PA deficit. (Odds ratio = 2.55) Total 12.30 ± 4.73 (*p* < 0.001) Onset 3.94 ± 1.83 (*p* = 0.004) Rhyme 4.52 ± 1.77 (n.s.) Tone 3.84 ± 2.36 (n.s.) **RAN test** 31.25% of DCD children have RAN deficit. (Odds ratio = 3.48) Total 65.91 s ± 16.06 (*p* < 0.001) Digit naming 45.62 s ± 16.06 (*p* < 0.001) Colour naming 78.23 s ± 33.99 (*p* < 0.001) Object naming 73.89 s ± 18.25 (*p* < 0.001)
Dyck and Piek (2010)	20/91	No clinical DCD diagnosis DSM‐IV criteria: A: M‐ABC Pc≤5 B: Referred children had motor issues affecting academics or daily activities, leading to specialised programs. C: Not assessed D: No physical disorders likely to affect physical performance were reported. 2 children with co‐occurring ADHD were excluded	8.55y (5y0m to 13y1m) (still including 2 with ADHD)	**CELF** (Mean SS 100 ± 15) Receptive Language. Mean SS poor motor coordination group = 95.9 ± 17.7 Expressive language. Mean SS poor motor coordination group = 102.6 ± 10.8	*DCD children have sig. worse ‘receptive language’ and ‘expressive language’ compared to children with poor motor coordination (but not DCD). However, only the ‘Receptive language’ mean score is below average*. **CELF** Receptive language mean SS DCD = 81.8 ± 21.9 (*p* < 0.05) Expressive Language mean SS DCD = 90.1 ± 21.6 (*p* < 0.05)
Farmer et al. (2016)	129/165	Clinical DCD diagnosis DSM‐V criteria: A: Not assessed B: DCDQ C: Not assessed D: DCD‐Clinic‐assessed children were excluded if showing pervasive development signs, IQ <70 (except for 5 scoring 70–85), abnormal MRI, neurological pathologies beyond DCD, abnormal EEG, significant prematurity, Cerebral Palsy, or genetic syndromes. 61 children with DCD were also treated for ADHD	8.8y ± 2.8y (4–8y)	Assessment by speech therapist and/or tested by neurological examination.	*DCD children often had an additional diagnosis of orofacial and/or verbal dyspraxia*. 46/129 (36%) were diagnosed with orofacial dyspraxia. 33/129 (26%) were diagnosed with verbal dyspraxia.
Flapper and Schoemaker (2013)	40/40	Clinical DCD diagnosis DSM‐IV criteria: A: M‐ABC≤ Pc 15 B: BHK Writing test C: not assessed D: IQ <70 was excluded (WISC‐R). Mean IQ was 88.7 ± 11.6. Children with PDD‐NOS were excluded.	5–13y	**TVK** (Norm: 5% < average) Auditory discrimination Auditory Synthesis Vocabulary production Sentence Structure Production of Word Forms Assessment of Word Forms Production sentence structure Test for concealed meaning Vocabulary choice test Word recognition test	*DCD children more often difficulties with ‘Auditory Discrimination’ (17% compared to 5%) and ‘Synthesis’ (8% compared to 5%) but no other domains*. **TVK** Auditory Discrimination: 17% < average Auditory Synthesis: 8% < average Other domains: relatively few difficulties
Gaines et al. (2008)	5/8	Clinical DCD diagnosis DSM‐IV criteria: A: BOT or M‐ABC B: Interview C: Not assessed E: IQ was measured. All scored within average zone. Parents had a college or university degree. 1 child had co‐occurring ADHD.	6.4y (only children; (0–13 y)	**CELF‐4** (mean SS 100 ± 15) **CTOPP** (mean SS 100 ± 15) Phonological awareness Phonological memory Rapid naming **GFTA‐2** (Pc)	*There was no evidence of lower scores in CELF or CTOPP. Only 1 out of 3 participants had a below average score on ‘Rapid Naming’. DCD participants often scored below or borderline average on the GFTA‐2*. **CELF‐4** SS per DCD participant = 126, 114, 96, and 102 **CTOPP** Phonological awareness SS per DCD participant = 97, 112, 104 Phonological memory: SS per DCD participant = 109, 94, 85 Rapid naming: SS per DCD participant = 100, 94, 52 **GFTA‐2** Pc per DCD participant = 2, 18, 6 and 20
Ho and Wilmut (2010)	5/10	Clinical DCD diagnosis DSM‐IV criteria: A: M‐ABC‐1 was performed but not used as inclusion criterion. All scored ≤Pc2. TD age‐matched all scored between Pc 20–70. B: Not assessed C: Not assessed D: Not assessed but IQ was measured (Range DCD WISC 85 ‐ 122.)	11y (9–13y)	Vicon motion capture system tracked movement of 4 markers (middle forehead, upper lip, lower lip, and chin). Children were asked to produce different sounds by example. **Non‐verbal open‐close in TD** Mean duration of movement TD at normal speed (853 ms ± 107) and fast speed (413 ms ± 127). Mean extent of movement between lips at normal speed (33.4 mm ± 4.4) and fast speed (24.5 mm ± 2.6) **Single words in TD** Mean duration of movement = 616 ms ± 116. Mean extent of movement between lips = 7.8 mm ± 1.9. **Monosyllable in TD** papapa/bababa: Overall duration = 12.52 s ± 2.24. *n* of syllables produced = 48.0 ± 9.6. tatata/dadada: Overall duration = 11.42 s ± 2.61. *n* of syllables produced = 37.0 ± 10.3. kakaka/gagaga: Overall duration = 11.57 s ± 1.87. *n* of syllables produced = 38.9 ± 11.9. **Tri‐syllable in TD** Overall duration = 13.59 s ± 1.20. *n* of syllables produced = 36.5 ± 4.4.	*No differences for non‐verbal movements or for single syllable words. At low task demands (open‐close movements, 1‐syllable words, and self‐paced speech), children with DCD at best showed patterns of performance indistinguishable from the control group and at worst showed mild spatial and temporal shortened movements. On more complex sentence repetition tasks and higher task demands (faster production), children with DCD showed shortened movements in extent and duration compared to TD*. **Non‐verbal open‐close in DCD**: Mean duration of movement at normal speed (829 ms ± 359; n.s.) and fast speed (506 ms ± 304; n.s.). Mean extent of movement between lips at normal speed (29.0 mm ± 4.1; n.s.) and fast speed: 23.6 mm ± 6.3; n.s.). **Single words in DCD**: Mean duration of movement: 581 ms ± 52 (n.s.). Mean extent of movement between lips: 6.7 mm ± 2.6 (n.s.). **Monosyllable in DCD**: papapa/bababa: Overall duration = 8.69 s ± 1.39 (*p* = 0.044). *n* of syllables produced = 34.9 ± 10.5 (*p* = 0.028) tatata/dadada: Overall duration = 8.73 s ± 2.03. *n* of syllables produced = 23.9 ± 5.2 kakaka/gagaga: Overall duration = 8.07 s ± 2.35. *n* of syllables produced = 26.4 ± 11.0 **Tri‐syllable**: Overall duration = 10.49 s ± 2.40 (*p* = 0.019). *n* of syllables produced = 27.9 ± 4.8 (*p* = 0.031)
King‐Dowling et al. (2015)	37/214	No clinical DCD diagnosis DSM‐V criteria: A: M‐ABC‐2 Pc≤16 B: not assessed C: not assessed D: Children with known physical impairments (e.g. blindness, deafness, or genetic syndromes) were excluded. Mean IQ with Kaufman Brief Intelligence Test 2 pDCD = 95.1, mean in TD = 98.9	59m (44–80m)	**PLS‐4** Total Language Pc 81.8 Auditory Comprehension Pc 79.5 Expressive Communication Pc 78.7	DCD have sig. worse performance on all PLS‐4 measures, yet all scores are within‐average. **PLS‐4** Total Language Pc 70.5 (*p* = 0.006) Auditory Comprehension Pc 69.9 (*p* = 0.006) Expressive Communication Pc 67.6 (*p* = 0.008)
Lingam et al. (2010)	346/6902	No clinical DCD diagnosis DSM‐IV criteria: A: ALSPAC Coordination test Pc <15 B: Failed Key Stage 1 writing test or pc<15 on parental ADL questionnaire C: not assessed D: Exclusion children with visual deficits, neurologic conditions like Cerebral Palsy, or an IQ <70 (Mean WISC‐III DCD group = 93.90 ± 16.40).	7–9y	**WOLD** Oral Expression Language Comprehension	*Children with DCD have increased odds for difficulties with ‘Oral Expression’ and ‘Language comprehension’*. **WOLD** Oral Expression: OR 3.18 [2.22–4.57] (*p* < 0.001) Language Comprehension: OR 2.43 [1.62 ‐ 3.64] (*p* < 0.001)
Mirabella et al. (2017)	18/36	Clinical DCD diagnosis DSM‐V criteria: A: Neuromotor status and standardised assessments with M‐ABC total pc ≤pc 15 or pc≤ 5 of one of the subtests B: Family history, developmental and medical history. DCD‐Q suspect or indicative of DCD C: not assessed D: WISC near normal intellectual abilities, PPVT score ≥85 (mean WISC in DCD group = 104.7 ± 19.1). Exclusion: neurological (Cerebral Palsy) or psychiatric disorders (ASD, ADHD). Children with learning and language disorders were excluded as well as left‐handed children.	DCD 9.87y ± 1.46 (7 ‐ 12.1y) TD 10y ± 0.7 (7.5 ‐ 11y)	**Reaction time semantic task** TD: sig. faster when arm/hand related verbs compared to leg/foot related verbs (Arm/hand related verbs Mean TD = 390 ± 44; Leg/foot related verbs: Mean TD = 371 ± 44) ***n* errors in semantic task**: Mean TD = 9.52 ± 4.86 **Reaction time colour discrimination task** TD sig. faster when leg/foot related verbs compared to abstract related verbs (Arm/hand related verbs: Mean TD = not reported; Abstract‐related words: Mean TD = 355 ± 32; Leg/foot related verbs = Mean TD = 339 ± 28) ***n* errors in colour discrimination task** Mean TD = not reported	*Unlike TD children who are faster in semantic tasks when arm/hand related verbs are used compared to leg/foot related terms, there is no difference in reaction time between arm/hand and leg/foot related verbs in DCD children. Additionally, there is no difference between arm/hand or leg/foot and abstract/related verbs in colour discrimination tasks in DCD children while TD children are sig. faster when leg/foot related verbs are used compared to abstract related verbs. Children with DCD tend to make sig. more errors in semantic tasks, but not in colour discrimination tasks*. **Reaction time semantic task** DCD: no sig. diff between arm/hand and leg/foot (Arm/hand related verbs: Mean DCD = 389 ± 56; Leg /foot related verbs: Mean DCD = 385 ± 55) ***n* errors in semantic task** Mean DCD = 16.96 (10.09) (*p* = 0.008) **Reaction time colour discrimination task** DCD no sig. difference between arm/hand or leg/foot and abstract/related (Arm/hand related verbs: Mean DCD = 357 ± 50; Abstract‐related words: Mean DCD = 351 ± 48; Leg/foot related verbs: Mean DCD = 355 ± 53) ***n* errors in colour discrimination task** Mean DCD = not reported; DCD no sig. diff. to TD
Rodger et al. (2007)	60/60	Clinical DCD diagnosis DSM‐IV criteria: A: Significant motor deficit on the NDPA (Neuro‐developmental Physiotherapy Assessments) B: Parental report that motor difficulties affected functioning. Children were referred for motor problems. C: / D: No known sensory, motor, neurological or intellectual impairment (all were in regular schools).	72.45m (52–95m)	**CELF‐3** (mean SS 100 ± 15) **CELF‐P** (mean SS 100 ± 15) **BST** (1–4, normal to severe rating) **VMPAC** (1–4 meaning normal‐severe rating) Global motor (oral reflexes) Focal (Oral‐motor production) Sequencing **GFTA** (Pc)	*Children with DCD scored within average on the CELF and the BST. Children with DCD show sig. poorer results on ‘Global Motor’ and ‘Focal’, but not ‘sequencing’, than expected for children that age. They scored average on the GFTA*. **CELF‐3/CELF‐P** DCD mean SS = 103.0 ± 13.8 **BST** DCD mean SS = 1.3 ± 0.9 (= normal score) **VMPAC** Global motor 3.3 ± 1.1 (= moderate dysfunction) Focal 2.8 ± 1.3 (= mild‐moderate dysfunction) Sequencing 1.6 ± 0.9 (= normal to mild dysfunction) **GFTA** DCD mean Pc = Pc 35 ± 25
Wisdom et al. (2007)	22/82	No clinical DCD diagnosis DSM‐IV criteria: A: M‐ABC Pc≤5 B: Motor problems affect daily life. For this reason children were in special education or OT programs. C: not assessed D: No physical disorders likely to affect physical performance were reported. 2 children in DCD group had ADD.	8.55y ± 2.07 (5y0m ‐ 13y1m)	**CELF‐3** (mean 100, SD 15) Receptive language Expressive language	*DCD group performed below average in the domain ‘receptive language’. They scored within average in the domain of ‘Expressive language’*. **CELF‐3** Receptive language: 82.07 ± 21.07 Expressive language 90.06 ± 20.59

Abbreviations: AD(H)D, Attention Deficit Hyperactivity Disorder; ALSPAC, Avon Longitudinal Study of Parents and Children; ASD, Autism Spectrum Disorder; BHK, Beoordeling Handschriften van Kinderen; BNT, Boston Naming Test; BOT, Bruininks‐Oseretsky Test of Motor Proficiency; BPVS‐2, British Picture Vocabulary Scales; BST, Bus Story Test; C‐TONI, Comprehensive Test of Nonverbal Intelligence; CELF, Clinical Evaluation of Language Fundamentals; CELF‐P, Clinical Evaluation of Language Fundamentals Preschool; CNRep, Children's Test of Non‐word Repetition; CTOPP, Comprehensive Test of Phonological Processing; DCD, Developmental Coordination Disorder; DSLD, Developmental Speech Language Disorder; DSM, Diagnostic and Statistical Manual for Mental Disorders; EEG, Electroencephalogram; GFTA, Goldman‐Fristoe Test of Articulation; IQ, Intelligence Quotient; LCT, Linguistic Comprehension Test; M, Months; M‐ABC, Movement Assessment Battery for Children; Mm, Millimeters; MRI, Magnetic Resonance Imaging; Ms, Milliseconds; *N*, Number; n.s., Not Significant; Ns, Not Significant; OR, Odds Ratio; PAT(‐C), Phonological Awareness Test (Chinese version); Pc, Percentile; PDD‐NOS, Pervasive Developmental Disorder Not Otherwise Specified; PLS, Preschool Language Scales; RAN, Rapid Automatic Naming; ScSc, Scaled Score; SD, Standard Deviation; Sig, Significant(ly); SS, Standard Score; TD, Typically Developing; TROG, Test for Reception of Grammar; TVK, Taaltest Voor Kinderen; VMPAC, Verbal Motor Production Assessment for Children; WISC(‐R), Wechsler Intelligence Scale for Children (Revised edition); WOLD, Wechsler Objective Language Dimensions; Y, Year(s).

### 3.2 Study Outcomes

The most prevalent standardised assessment instruments were the Clinical Evaluation of Language Fundamentals (Archibald and Alloway 2008; Dyck and Piek 2010; Rodger et al. 2007; Wisdom et al. 2007), the Peabody Picture Vocabulary Test (Celletti et al. 2015; Cheng et al. 2009; Cheng et al. 2022), the Goldman‐Fristoe Test of Articulation (Archibald and Alloway 2008; Gaines et al. 2008; Rodger et al. 2007) and the Bus Story Test (Celletti et al. 2015; Rodger et al. 2007). Other assessment instruments were only used once: the Boston Naming test (Celletti et al. 2015), the British Picture Vocabulary Scales (Archibald and Alloway 2008), the Composite Speech/Language Tests (Cheng et al. 2009), the Comprehensive Test Of Phonological Processing (Gaines and Missiuna 2007), Language Ability Assessment for Preschoolers (Cheng et al. 2009), Linguistic Comprehension Test (Celletti et al. 2015), Memorial‐training test for the assessment of reading and comprehension skills (Celletti et al. 2015), Phonological Awareness Test (Cheng et al. 2022), the Preschool Language Scales (King‐Dowling et al. 2015), the Rapid Automatic Naming test (Cheng et al. 2022), Taaltest voor Kinderen (Flapper and Schoemaker 2013), the Test for Reception Of Grammar (Archibald and Alloway 2008), Wechsler Objective Language Dimensions (Lingam et al. 2010), the Children's Test of Nonword Repetition (Archibald and Alloway 2008), and the Verbal motor Production Assessment for Children (Rodger et al. 2007). Three studies included quantitative standardised measures of articulation rate (Archibald and Alloway 2008), reaction time and number of errors in different tasks (Mirabella et al. 2017), and kinematic oral motor analyses (Ho and Wilmut 2010). One study focused on identifying verbal and orofacial dyspraxia (Farmer et al. 2016).

### 3.3 Language Performance

Several studies have reported significant performance differences between children with DCD and those with poor motor coordination (but no DCD) or typically developing children (Archibald and Alloway 2008; Cheng et al. 2022; King‐Dowling et al. 2015; Dyck and Piek 2010). Although the mean performance of the DCD group was still within the average range, Celletti et al. (2015) reported a higher prevalence of DLD diagnoses among these children. Similarly, Cheng et al. (2009) found that children with DCD were three times more likely to be diagnosed with DLD or SSD, while Farmer et al. (2016) reported prevalence rates of 36% for orofacial dyspraxia and 26% for verbal dyspraxia within this group.

In evaluating language skills among children with DCD, five studies reported average performance in the domains of receptive vocabulary (Archibald and Alloway 2008; Cheng et al. 2022) and auditory comprehension (King‐Dowling et al. 2015). When receptive language was measured with Clinical Evaluation of Language Fundamentals, below‐average performance was observed in an overlapping sample (Wisdom et al. 2007; Dyck and Piek 2010). Language deficits were found in nonword repetition and expressive language (Lingam et al. 2010), sentence recall (Archibald and Alloway 2008) and narrative abilities (Celletti et al. 2015). Additionally, deficits have been documented in phonological awareness (Cheng et al. 2022) and auditory synthesis and discrimination (Flapper and Schoemaker 2013). Mirabella et al. (2017) reported differences in the processing times for action verbs between children with DCD and typically developing children. Children with DCD made significantly more errors on action‐related semantic tasks but not on colour discrimination tasks. In the manual go/no‐go test (react only when a specific stimulus is shown), TD children responded slower to arm‐related verbs than to leg‐related verbs (e.g., to chop or to run, respectively). This delay likely reflected increased motor cortex activation when both comprehending the verbs and executing manual movements involving the same body part. This effect was not observed in children with DCD.

Five articles showed average performance in the following areas: expressive language (Dyck and Piek 2010; Wisdom et al. 2007; King‐Dowling et al. 2015); story recalling (Rodger et al. 2007); rapid automatic naming (Cheng et al. 2022); word structure, non‐word repetition and grammatical competence (Archibald and Alloway 2008; Rodger et al. 2007).

### 3.4 Speech and Oral Motor Performance

The global and focal oral motor production measured by the Verbal Motor Production Assessment for Children was significantly poorer than expected for the children with DCD (Rodger et al. 2007). Slower articulation rates were found in one study (Archibald and Alloway 2008). On a kinematic level, Ho and Wilmut (2010) reported markedly different motor control in children with DCD but only in complex sentence repetition and fast production, even though the children with DCD who took part in the study showed no overt speech or language problems. Studies using Goldman–Fristoe Test of Articulation (GFTA) showed average speech production (Archibald and Alloway 2008; Gaines et al. 2008; Rodger et al. 2007). However, in the study by Rodger et al. (2007), nearly 50% of the participants exhibited sound distortion.

## 4 Discussion

This study aimed to explore the relation between (p)DCD and speech, language and oral motor performance by evaluating fourteen articles. While the limited number of available studies applied heterogeneous standardised instruments, preventing definitive conclusions, the results carefully suggest heightened difficulties in children with DCD across various language domains, including expressive and receptive language, auditory discrimination and synthesis, as well as speech and oral motor abilities.

The most consistent finding across studies was the relationship **between (p)DCD and both speech production and oral motor development**, with children showing slower articulation rates (Cheng et al. 2022; Archibald and Alloway 2008), difficulties in verbal motor production (Rodger et al. 2007), and a high prevalence of verbal and orofacial dyspraxia diagnoses (potential diagnosis of childhood apraxia of speech) (Farmer et al. 2016). However, when the children's speech production was assessed using the Goldman–Fristoe Test of Articulation, a commonly used diagnostic tool for speech disorders (Skahan et al. 2007), performances were generally within the average range (Rodger et al. 2007; Archibald and Alloway 2008; Gaines et al. 2008). However, Gaines et al. (2008) suggested the presence of possible phonological or articulatory challenges in some individuals with DCD and Rodger et al. (2007) reported a high prevalence of sound distortion. Interestingly, Ho and Wilmut (2010) reported that differences between children with DCD and TD peers became more apparent as speech production complexity increased, with children with DCD demonstrating atypical oral‐motor patterns during complex speech tasks that were not observed in simpler productions.

Studies examining language difficulties have found a high prevalence of diagnosed language disorders among children with DCD (Cheng et al. 2009; Celletti et al. 2015). Several high quality studies have reported significant deficits in **receptive language** abilities in children with DCD compared to control groups (Cheng et al. 2009; Cheng et al. 2022; Wisdom et al. 2007; King‐Dowling et al. 2015; Lingam et al. 2010; Dyck and Piek 2010), whereas some lower quality studies found no such differences (Archibald and Alloway 2008; Gaines et al. 2008; Rodger et al. 2007). Further, the narrative difficulties in children with DCD (Celletti et al. 2015), slower non‐word repetition (Archibald and Alloway 2008), may suggest **expressive language difficulties** (Lingam et al. 2010), although several studies found within‐average results on expressive language tests (Dyck and Piek 2010; Wisdom et al. 2007; King‐Dowling et al. 2015; Rodger et al. 2007; Cheng et al. 2022; Archibald and Alloway 2008). These studies focused primarily on grammar, vocabulary, and narrative language and did not assess the children's speech or oral motor abilities. This limited focus may contribute to an underestimation of SSD in this population. Moreover, none of the speech or language‐focused studies evaluated oral motor skills, leaving it unclear whether the children had motor difficulties affecting articulation or linguistic difficulties affecting phonology and their expressive language abilities (Stringer et al. 2024). According to the embodied theory of language (Gallese 2007), which posits that understanding action‐related words involves motor system activation, findings from Mirabella et al. (2017) support the hypothesis that, unlike TD children, individuals with DCD may exhibit reduced motor cortex activation during the processing of action‐related words. This reduced activation may contribute to weaker comprehension of action‐related language. However, future research employing neuroimaging techniques is necessary to validate this hypothesis.

Although clinicians and parents have observed early feeding challenges (Pless et al. 2001; Gurevitz 2019; Hitchcock et al. 2020), which have been proposed as potential precursors to later diagnoses of DCD (Rinat et al. 2022; Gaines and Missiuna 2007; Miniscalco et al. 2006), the current body of literature lacks the use of standardised assessments to comprehensively investigate this aspect. While language and speech difficulties may evolve throughout childhood and become more subtle over time, many parents report difficulties regarding articulation, speech, or language during the preschool years (De Roubaix et al. 2023; Pless et al. 2001), while others report advanced language skills even from a very young age (Missiuna et al. 2005). The result from our review suggests that the GFTA may not adequately detect the speech difficulties in children with DCD, as their difficulties may be more subtle or only emerge during more complex speech tasks, as shown by Ho and Wilmut (2010). Additionally, factors such as low self‐esteem associated with DCD (Omer et al. 2019) may contribute to the lower intelligibility perceived by parents. This variability in language development highlights the need for further research to better understand the developmental trajectories of language abilities in children with (p)DCD.

Although several studies report that children with DCD perform within normative ranges on speech and language assessments, their abilities often fall at a borderline level. The mean performance scores in these groups may be negatively skewed by poorer performance from a subset of individuals, suggesting that a significant proportion of children with DCD face challenges in speech, language, and oral motor skills, as confirmed by three included studies (Celletti et al. 2015; Cheng et al. 2009; Farmer et al. 2016). It is important to avoid viewing children with DCD as a homogeneous population, particularly given the high prevalence of co‐occurring conditions. Similarly, there is a higher prevalence of DCD among children with DLD and SDS (Celletti et al. 2015; Farmer et al. 2016; Hill 2001; Finlay and McPhillips 2013), indicating potential shared genetic factors (Gidziela et al. 2023). Recognising these co‐occurrences is crucial, as they can intensify difficulties and contribute to mental health challenges (Flapper and Schoemaker 2013; Farmer et al. 2016; Cranwell et al. 2015; Larson et al. 2011; Clegg et al. 2004; Lewis et al. 2021).

### 4.1 Strengths and Limitations

In this study, we specifically focused on language, speech, and oral motor difficulties in children with DCD. To enhance clarity, we opted to exclude studies examining motor difficulties in children with SLDs, recognising that this decision provides only one perspective on the overall picture. Furthermore, our inclusion criteria included solely standardised assessments, excluding subjective parental and/or clinical reports. While this enhances the robustness of our findings, it also imposes limitations on capturing a comprehensive overview. A key strength of our study is the interpretation of the mean performances rather than just differences between DCD and TD, which often proves more clinically relevant. Additionally, half of the included studies encompassed a group of clinically diagnosed children with DCD, enhancing generalizability to real‐life clinical practice. Yet we recognise the suggestive nature of the evidence as only a limited number of studies were available for analysis in this systematic review.

### 4.2 Conclusion

More high‐quality research is necessary to examine the prevalence of language, speech, and oral motor difficulties in children with DCD. Longitudinal studies could improve our understanding of the interplay between motor development and speech‐language development. The results of this review carefully suggest heightened difficulties in children with DCD across various language domains, as well as speech and, foremost, oral motor abilities.

## Conflicts of Interest

The authors report no conflict of interest.

## Data Availability

The data that support the findings of this study are available from the corresponding author upon reasonable request.
